# Cone-Beam CT Segmentation for Intraoperative Electron Radiotherapy Based on U-Net Variants with Transformer and Extended LSTM Approaches

**DOI:** 10.3390/cancers17030485

**Published:** 2025-02-01

**Authors:** Sara Vockner, Matthias Mattke, Ivan M. Messner, Christoph Gaisberger, Franz Zehentmayr, Klarissa Ellmauer, Elvis Ruznic, Josef Karner, Gerd Fastner, Roland Reitsamer, Falk Roeder, Markus Stana

**Affiliations:** 1 Department of Radiation Therapy and Radiation Oncology, Paracelsus Medical University, 5020 Salzburg, Austria; m.mattke@salk.at (M.M.); i.messner@salk.at (I.M.M.); c.gaisberger@salk.at (C.G.); f.zehentmayr@salk.at (F.Z.); k.ellmauer@salk.at (K.E.); e.ruznic@salk.at (E.R.); j.karner@salk.at (J.K.); g.fastner@salk.at (G.F.); f.roeder@salk.at (F.R.); m.stana@salk.at (M.S.); 2 Institute of Research and Development of Advanced Radiation Technologies (radART), Paracelsus Medical University, 5020 Salzburg, Austria; 3 Department of Gynecology, Paracelsus Medical University, 5020 Salzburg, Austria; r.reitsamer@salk.at

**Keywords:** deep learning, automatic segmentation, intraoperative electron radiotherapy, cone beam computed tomography

## Abstract

Artificial Intelligence (AI) applications are increasingly prevalent in radiotherapy, including commercial software solutions for automatic segmentation of anatomical structures for 3D Computed Tomography (CT). However, their use in intraoperative electron radiotherapy (IOERT) remains limited. In particular, no AI solution is available for contouring cone beam CT (CBCT) images acquired with a mobile CBCT device. The U-Net convolutional neural network architecture has gained huge success for medical image segmentation but still has difficulties capturing the global context. To increase the accuracy in CBCT segmentation for IOERT, three different AI architectures were trained and evaluated. The features of the natural language processing models Transformer and xLSTM were added to the popular U-Net architecture and compared with the standard U-Net and manual segmentation performance. These networks were trained and tested using 55 CBCT scans obtained from breast cancer patients undergoing IOERT in the department of radiotherapy and radiation oncology in Salzburg, and each architecture’s segmentation performance was assessed using the dice coefficient (DSC) as a similarity measure. The average DSC values were 0.83 for the standard U-Net, 0.88 for the U-Net with transformer features, and 0.66 for the U-Net with xLSTM. The hybrid U-Net architecture, including Transformer features, achieved the best segmentation accuracy, demonstrating an improvement of 5% on average over the standard U-Net, while the U-Net with xLSTM showed inferior performance compared to the standard U-Net. With the help of automatic contouring, synthetic CT images can be generated, and IOERT challenges related to the time-consuming nature of 3D image-based treatment planning can be addressed.

## Simple Summary

This study explores the use of advanced neural network architectures to automatically segment CBCT images acquired during intraoperative electron radiotherapy (IOERT). By integrating self-attention and xLSTM features with the U-Net architecture, improved segmentation accuracy for relevant anatomical structures was achieved. These results pave the way for the optimisation of IOERT workflows and facilitate the generation of synthetic CT images. This is a basis for adaptive 3D treatment planning, ultimately enhancing treatment precision in IOERT.

## 1. Introduction

Intraoperative Electron Radiotherapy (IOERT) complements External Beam Radiotherapy (EBRT) in cancer treatment by delivering radiation directly to the tumour bed during surgery. This approach allows for precise tumour bed localisation and sparing of organs at risk, enabling delivery of high-dose fractions [1]. IOERT is an established technique for partial breast irradiation and has been investigated in the context of large prospective studies [2,3]. While dose calculation accuracy in EBRT planning has benefited significantly from 3D voxel-based patient models using imaging modalities such as Computed Tomography (CT), IOERT workflows mostly rely on a 2D ultrasound and tabulated dose calculation based on energy absorption in water [4]. The adoption of a 3D dose calculation in an IOERT setting remains limited because of time constraints and the complexity of the workflow. Mobile Cone Beam CT (CBCT) devices, such as the ImagingRing-m (medPhoton, Salzburg, Austria), provide the opportunity to integrate 3D imaging during surgery [5]. A treatment planning software with Monte Carlo Dose calculation dedicated to electron-beam IOERT is available under the name Radiance TPS (GMV, Spain) [6]. Even though geometrical mismatches can be avoided using this software [5], it is not yet applicable to in-room imaging in routine clinical practice [7]. The missing piece is the segmentation of image data not only for the accurate delineation of organs at risk and target volumes but also to allow for a Dose Volume Histogram (DVH)-based plan optimization and even more for the generation of a synthetic CT to address issues related to Hounsfield Units (HU) uncertainties due to the image quality of CBCTs. As compared to a standard planning CT for EBRT, images from a mobile CBCT have lower image quality. This is due to uncertainties in the HUs influenced by the imaging protocol, scatter contribution, and artefacts, as discussed by Hensley in this context [8]. Additional artefacts can be caused by the electron applicator or a metallic protection plate. Manual segmentation of CBCT data is not feasible in the time frame of an intraoperative setting. Even though commercial AI solutions for automatic segmentation exist, they are not applicable to CBCT data from devices like the ImagingRing-m. This is due to technical challenges like artefacts that degrade the pixel intensity consistency, which is critical for training segmentation models [9]. This inconsistency affects the ability of neural networks to learn robust and generalised features and differentiate fine anatomical structures [10]. Additionally, the limited field of view exacerbates these challenges, as neural networks rely heavily on spatial and contextual information to make accurate segmentations [11]. To address these limitations, this study explores the implementation, training, and evaluation of three advanced neural network architectures to automatically segment the ribs, the lung, the applicator of the linear accelerator (linac), and the tissue located within the applicator on CBCT images that were acquired during surgery.

## 2. Materials and Methods

At the Department of Radiotherapy and Radiation Oncology, Salzburg, Austria, an anticipated intraoperative electron radiation therapy (IOERT) during breast-conserving surgery followed by conventional whole breast irradiation is established as the preferred boost technique to the tumour bed [12]. Following the surgical removal of the tumour, a fully X-ray-compatible POM-C applicator is positioned around the surgically adapted tumour bed. To verify that the applicator is accurately positioned, a CBCT image is acquired in the treatment position on the X-ray-compatible operating table without moving the patient [5]. Based on the acquired CBCT image, the applicator position can be adjusted if necessary; geometrical information about tissue thickness relevant to treatment planning is retrieved, and radiation treatment planning can be performed.

The acquisition protocols used a tube acceleration voltage of 120 kVp and an additional filtration of 0.5 mm Cu. The trajectory design, dose, and reconstruction parameters were tuned iteratively to account for varying factors, such as field of view geometry, surgical equipment placement, and patient size [13]. The FOV was defined during the volume definition workflow [14], with its centre positioned near the patient side of the applicator, ensuring that the FOV size encompassed both the applicator opening and the rib cage. As a result, all scans were conducted at an off-axis imaging centre from the gantry isocentre with patient-specific variations in isocenter distance and FOV size. The resulting images were suitable for radiation dose calculation and AI training, facilitated by automated scaling that includes heuristic corrections for object and head scatter, as well as beam hardening [5].

### 2.1. Neural Network Architectures

In this study, we investigated three different neural network architectures for the segmentation of CBCT images: U-Net, Transformer, and xLSTM. The first model utilised was the U-Net architecture, which is well-established in the field of medical imaging. It is well-suited for clinical applications because of its ability to perform object classification and detection with limited training data. Characteristic is its symmetric U-shaped design, consisting of an encoder on one side and a decoder on the other side. The encoder compresses the input image into an abstract representation by extracting relevant features, while the decoder reconstructs these features into a segmentation map. Skip connections between the encoder and decoder allow precise localisation by transferring high-resolution features directly, thus preserving fine-grained details and anatomical boundaries [15].

The second architecture we investigated was a Transformer-based model inspired by its success in natural language processing tasks. Transformers are highly effective at capturing long-range dependencies and global context, which can be very important for understanding complex anatomical structures in medical images. The core component of the Transformer is the Self-Attention (SA) mechanism, which allows the model to weigh the importance of each element in relation to all others in the input sequence [16]. In the context of medical image segmentation, this mechanism helps the model effectively capture dependencies between distant anatomical structures, leading to an improved understanding of spatial relationships and better segmentation accuracy. However, a significant limitation of Transformer networks is their high computational complexity and memory requirements, particularly for large inputs. Moreover, Transformers typically require large amounts of training data to perform well, which can be a constraint in clinical settings with limited data availability [17].

The third architecture employed was an adaptation of the original long short-term memory (LSTM) network, known as xLSTM. LSTMs are effective at capturing long-term dependencies in sequential data because of their memory cell structure [18]. The xLSTM variant incorporates several improvements, such as increased parallelisation, enhanced gating mechanisms, and improved gradient flow, enabling it to compete with Transformers in both scalability and performance [19].

In this study, we aimed to combine the strengths of U-Net, Transformer, and xLSTM architectures to enhance segmentation accuracy for CBCT scans acquired during intraoperative electron radiotherapy (IOERT) procedures. Specifically, we explored whether the ability of U-Net to capture fine-grained details, along with the capacity of Transformer and xLSTM architectures to model long-range dependencies, can complement each other in improving the segmentation of ROIs within the CBCT images. Additionally, because of limited training data and hardware constraints, we combined these architectures to develop a solution that is designed to achieve high segmentation accuracy without the need for large-scale computational resources.

#### 2.1.1. Self-Attention to U-Net

We extended the traditional U-Net architecture by integrating the Transformer’s self-attention mechanism to capture long-range dependencies within the feature maps. This modified network includes the encoding and decoding paths as per the original U-Net [15], but with additional self-attention [16] operations applied in the decoding path. The encoding path is based on the conventional U-Net structure, consisting of four blocks of consecutive convolutions, each followed by max pooling operations [15]. At the bottleneck layer, two convolutional operations with 1024 filters were used, each followed by a dropout layer to prevent overfitting. The decoding path mirrors the encoding process but with an upsampling operation to reconstruct the spatial resolution. The skip connections from the corresponding encoding layers are concatenated to help recover finer details lost during downsampling. In order to capture non-local interactions and dependencies, a self-attention block was added after the decoding path. Within the attention block, the feature map from the final decoding block is projected into query (*Q*), key (*K*), and value spaces using 1 × 1 convolutions. The attention matrix (*A*) is then created by the application of the softmax function [20] to the dot product of the query matrix and the transpose of the key matrix, multiplied with the value matrix (*V*), as presented in [16]:

$$A=softmax(Q\times K^{⊺})V.$$

Finally, a residual connection is applied, combining the attention output *A*, scaled by a factor γ, with the original input.

#### 2.1.2. xLSTM to U-Net

Additionally, we trained a 2D encoder model architecture based on the xLSTM-U-Net, as described in [21]. This model architecture follows a conventional U-Net structure, incorporating xLSTM blocks within multiple encoder layers to capture both local features and long-range dependencies. Each encoder layer contains consecutive residual blocks [22], involving convolution and Instance Normalisation (IN) [23], followed by an xLSTM block, as described in [18]. After feature extraction, the reshaped outputs of the xLSTM blocks are concatenated into the decoding process for the generation of the segmentation mask. The decoder consists of residual blocks and transposed convolutions and leverages skip connections from the encoder part.

### 2.2. Dataset and Data Pre-Processing

The CBCT device ImagingRing-m was implemented in the clinical routine workflow for IOERT procedures in our hospital in 2022. The CBCTs used in this study were acquired from patients treated routinely between 18 May 2022 and 1 August 2024 and used retrospectively for this analysis. The study was approved by the local ethics committee of the Federal State of Salzburg with the approval number 1096/2024. The dataset consists of 55 female breast cancer patients receiving an IOERT boost. Patient selection for boost irradiation was performed according to current national guidelines [24,25]. Patients receiving IOERT treatment as a 21 Gy single shot were excluded. In these cases, a protection plate would be needed [12], which would introduce additional artefacts. Also, patients with low tissue volume, which gives the need for the application of a bolus disc for dosimetric reasons, were excluded, as this would bias the segmentation of the protruding tissue. The dataset consists of 29 patients receiving treatment for the left breast and 26 patients receiving treatment for the right breast. As patient data were anonymised before this particular analysis, no further details on patient characteristics can be listed. Because of our in-house IOERT regulations, all patients were at least 18 years old, and informed consent for the procedure was obtained beforehand. This CBCT dataset with annotated anatomical structures is currently, to our knowledge, the largest one published for breast IOERT. Since the numbers of IOERTs for other anatomic sites (e.g., extremities, retroperitoneum) are significantly smaller, these were not included in this study. On average, each patient’s CBCT dataset consists of 164 two-dimensional (2D) slices forming a three-dimensional (3D) CBCT image in the transversal plane. The CBCT images of each patient vary in size in the coronal and sagittal planes, as the field of view was individually determined for each patient during surgery. To create the annotations required for training of the CBCT datasets, anatomical structures such as the lungs, ribs, and the tissue protruding into the applicator, as well as the POM-C applicator itself, were manually contoured by clinical experts using the treatment planning software RayStation (RaySearch Laboratories, Stockholm, Sweden). The CBCT images and corresponding structures were subsequently exported in standard medical data formats (DICOM) for further processing in a programming environment. Because of the variability in the field of view across different CBCT images, data pre-processing involved padding the 2D CBCT slices to standardise their dimensions. For the U-Net and self-attention U-Net architectures, which rely on MaxPooling and Conv2DTranspose operations, input dimensions must be divisible by 2 at each down-sampling and up-sampling stage. Therefore, padding was applied to ensure that these requirements were met for effective feature extraction and reconstruction. In contrast, the U-Net+xLSTM architecture, which employs xLSTM blocks and adaptive feature processing, can accommodate non-standard input sizes without requiring strict down-sampling constraints. As a result, the 2D CBCT slices were padded and cropped to match the smallest possible dimension, optimising input compatibility while minimising information loss. This flexibility made U-Net+xLSTM suitable for handling the diverse CBCT data in this study.

### 2.3. Model Training

All three models were trained using an NVIDIA Quadro M6000 GPU with 24 GB of memory and CUDA version 11.8. Key training parameters for each model are summarised in Table 1. Because of the limited GPU performance, a batch size of 1 was used. In addition to the loss function, the Dice Similarity Coefficient (DSC) [26] was computed after each training step to evaluate the overlap between the annotated and predicted segmentation maps. The DSC between two sets is defined as

$$DSC\left(A,B\right)=2\frac{\left|A\cap B\right|}{\left|A|+|B\right|}.$$

Additionally, performance metrics—sensitivity, specificity, and precision—were used to evaluate the segmentation on the CBCTs. All metrics can be calculated using the four prediction outcomes: true positives (TP), false positives (FP), true negatives (TN), and false negatives (FN). Sensitivity, also called recall, measures the proportion of actual positives (correctly segmented regions) that are correctly identified by the model. It is defined as

$$Sensitivity=Recall=\frac{TP}{TP+FN}$$

Higher sensitivity indicates that the model effectively identifies the positive cases. A lower sensitivity suggests that anatomical details of the segmented ROI may be missing (under-segmentation) and a part of a relevant organ at risk may be exposed to more radiation than intended. Specificity measures the proportion of actual negatives (excluded regions) that are correctly identified. It is defined as

$$Specificity=\frac{TN}{TN+FP}$$

High specificity ensures that the model minimises false positives, which is critical in avoiding over-segmentation. Within a clinical setting, an over-segmentation of organs at risk can eventually lead to compromised dose coverage of the target volume. Precision measures the proportion of correctly identified positive predictions out of all positive predictions made by the model, indicating its ability to avoid false positives. This ensures accurate segmentation of the ROIs by the model. Precision is defined as [27,28,29]

$$Precision=2\cdot \frac{TP}{TP+FP}$$

For the training step, the dataset was randomly split into training (65%) and validation (16%) subsets. The three models were trained on the training set and evaluated on the validation set after each training epoch. Additionally, an independent test set (18%) consisting of ten patients was used for final evaluation. To address the potential risk of overfitting due to the limited dataset size and the complexity of hybrid architectures incorporating Transformer and xLSTM features, we implemented mitigation strategies, such as data augmentation, dropout regularisation, and careful hyperparameter tuning [30].

### 2.4. Transfer Learning

During the initial training phase, we observed that both the Transformer and U-Net models showed high loss values and low DSCs. The models were unable to effectively learn meaningful features. This was likely due to the limited quality of the CBCT data, which included imaging artefacts. Also, the need for significant padding resulted in the loss of critical information, influencing the performance. To address this issue, we applied transfer learning to all three models to improve the training process. As explained by Hosna et al. [31], transfer learning involves using pre-trained weights from a model that has already learned feature representations in a similar domain, which enables efficient training of a new model for a related task. This approach can be beneficial when dealing with limited training data, as it provides a solid foundation of learned features that can be further fine-tuned for the new problem. By utilising these pre-trained weights, the learning process becomes more efficient, leading to faster convergence and improved performance, especially in scenarios where the data quality or quantity is limited. In this case, a pre-trained model was utilised as the starting point, allowing the U-Net and U-Net+SA mechanism models to build upon previously learned features. To apply transfer learning, we trained a model for every architecture to automatically segment heart structures on CT images. The dataset consists of 85 annotated high-quality planning CT images with a fixed field of view, of which 10 were reserved for testing. With this approach, we were able to significantly improve the learning capability of those two models on the CBCT data. The U-Net+xLSTM model was not affected by the lack of pre-training, suggesting that its architecture was inherently allowed to learn effectively even without transfer learning.

## 3. Results

In this section, we present the evaluation results of the three neural network architectures for the segmentation of a test dataset including 10 CBCT images from breast cancer patients undergoing IOERT. The evaluation focused on the segmentation accuracy across four regions of interest (ROIs): ribs, lung, linac applicator, and the tissue protruding into the applicator. The performance of the segmentation is determined by the DSC between the manual segmentation (ground truth) and the predicted segmentation of one of the models.

**Figure 1:**
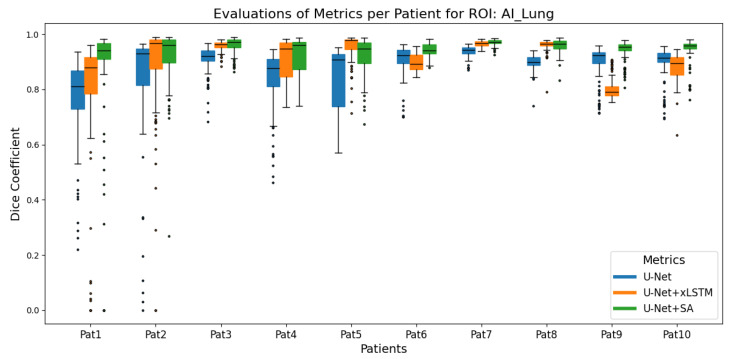
Comparison of DSCs of the lung ROI for the three model architectures, U-Net, U-Net+xLSTM, and U-Net+SA, for ten patients. U-Net+SA had the best performance, followed by U-Net (*p* = 0.002), while U-Net+xLSTM performed lowest with no statistically significant difference to U-Net.

**Figure 2:**
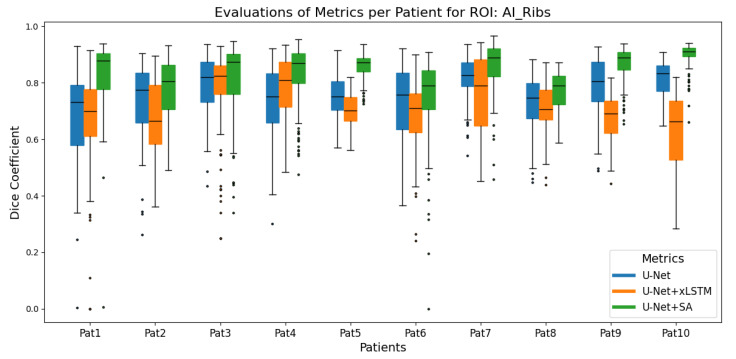
Comparison of DSCs of the ROI of ribs for the three model architectures, U-Net, U-Net+xLSTM and U-Net+SA, for ten patients. U-Net+SA had the best performance, followed by U-Net (*p* = 0.002), while U-Net+xLSTM achieved the lowest average DSC with a significant difference to U-Net (*p* = 0.049).

Box plots were used to summarise the distribution of the DSCs between manual and predicted segmentation for each 2D slice of a patient across 10 test patients for each architecture. Figure 1 and Figure 2 show the box plots for the rib and the lung ROIs, respectively. Summary statistics (median, minimum, and maximum DSCs) for the linac applicator and protruding tissue ROIs are presented in Table 2 and Table 3, respectively. In addition, the Wilcoxon signed-rank test was conducted at a significance level of 0.05 to compare the median DSCs between U-Net and U-Net+SA, as well as between U-Net and the hybrid U-Net+xLSTM. The Wilcoxon signed-rank test is a non-parametric statistical test used when the data do not meet the assumptions of normality, making it suitable for comparing paired non-normally distributed data, such as the median DSC across patients [32].

Figure 1 shows that the U-Net+SA model consistently achieved higher DSC compared to both the U-Net+xLSTM and the standard U-Net for segmentation of the lung. Nevertheless, an average DSC greater than 0.9 was achieved by each model across the test dataset. The Wilcoxon signed-rank test revealed a significant difference between U-Net and U-Net+SA (*p* = 0.002) but no significant difference between U-Net and U-Net+xLSTM (*p* = 0.193).

The U-Net+SA outperformed the other models for segmentation of the ribs, as can be seen from Figure 2, with an average DSC of 0.86 over all 10 test patients. Significant differences were found between U-Net and U-Net+SA (*p* = 0.002) but also between U-Net and U-Net+xLSTM (*p* = 0.049), with the standard U-Net performing better than U-Net+xLSTM.

The segmentation of the tube and protruding tissue ROIs led to less uniform results, including some outliers for the U-Net and U-Net+xLSTM architectures. Therefore, these results are presented in tables to provide precise numerical outcomes for the median DSC, which is still often greater than 0.8. For the tube region, Table 2 and the Wilcoxon signed-rank test show significant differences between U-Net and U-Net+SA (*p* = 0.002), with U-Net as the second-best model and U-Net+xLSTM showing comparable but slightly lower performance. The U-Net+SA reached the highest DSC, with an average of 0.92.

For the protruding tissue region, Table 3 and statistical tests show no significant differences between U-Net (average DSC of 0.79) and U-Net+SA (average DSC of 0.77), indicating, with a p-value of 0.85, that both models performed similarly. However, there was a significant difference between U-Net and U-Net+xLSTM (*p* = 0.002), with U-Net performing better than the U-Net+xLSTM. Additionally, we included sensitivity, specificity, and precision as metrics for model performance. They are summarised in Table 4, including the standard deviations. The U-Net+SA models demonstrated consistently high performance across all metrics, particularly in the lung ROI, where the model achieved the highest average sensitivity (0.94) and precision (0.97). While U-Net models showed variable performance in terms of sensitivity and precision, except in the protruding tissue ROI with the highest precision of 0.89. Similarly, U-Net+xLSTM models exhibited variability, with higher values in the lung ROI (precision = 0.97) and reduced sensitivity and precision in the protruding tissue ROI.

**Figure 3:**
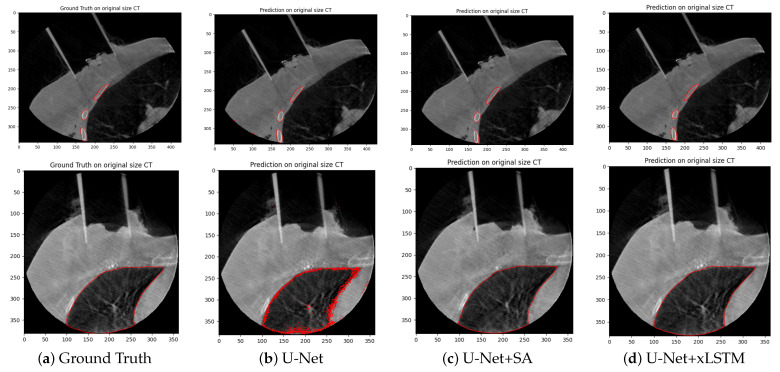
Two-dimensional segmentation of the ribs (**top**) and the lung (**bottom**) on CBCTs of the test dataset using the three network architectures on one representative image slice for one patient.

**Figure 4:**
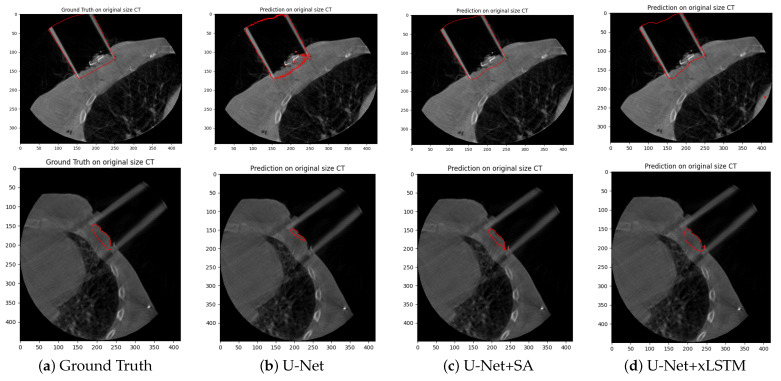
Two-dimensional segmentation of the applicator (**top**) and the protruding tissue (**bottom**) on CBCTS of the test dataset using the three network architectures.

Complementary to the quantitative evaluation, qualitative visualisations of the segmentation results are shown in the form of one representative axial slice of a patient in Figure 3 and Figure 4. These figures display the ground truth contours alongside the segmentation outputs from each of the three architectures. These images reveal that the U-Net+SA and U-Net+xLSTM architectures provide smoother and more anatomically consistent segmentation boundaries compared to the standard U-Net, particularly for structures with complex shapes, such as the lung and the applicator.

## 4. Discussion

The presented results indicate that neural network architectures can provide adequate segmentation of CBCT images essential to an adaptive 3D treatment planning procedure in IOERT, including, but not limited to, a foundation for the generation of synthetic CT images. Among the three tested architectures, the U-Net model incorporating self-attention (U-Net+SA) consistently outperformed the others across most ROIs, including the lung (on average, 4–5% higher DSC), ribs (on average, 8–13% higher DSC), the linac applicator (on average, 8–20% higher DSC), and the tissue (on average, 2–30% higher DSC), particularly after applying transfer learning. A previous study evaluating a commercial segmentation product reports that a Dice score above 0.8 is considered acceptable [33]. Given that our average DSC across all structures is 0.88 ± 0.09, our results can be considered to demonstrate a good level of segmentation performance, exceeding the threshold for acceptability. This suggests that the self-attention mechanism enhances the ability of the model to capture spatial features, especially in CBCT images with artefacts. Moreover, the use of transfer learning was crucial for improving the performance of both the U-Net and U-Net+SA models.

The U-Net+xLSTM model demonstrated variable performance across the ROIs. While it performed comparably to the other models for the ROIs of the lung and ribs, its outcomes were inferior for the protruding tissue in several cases, with lower DSC values. This performance variability highlights a key limitation of the xLSTM architecture in this application. The primary strength of xLSTM is its ability to process sequential or long-range data dependencies. This may explain its suboptimal performance compared to the self-attention-based U-Net architecture, which is better suited for capturing spatial dependencies. The model’s ability to perform without requiring transfer learning can be attributed to its advanced gating mechanisms, which allow it to capture local and some long-range dependencies [19]. However, this architectural feature did not translate into consistent improvements across all ROIs. Specifically, its performance in the tube and protruding tissue regions suggests that further optimisation or task-specific modifications are required to improve its applicability. These findings suggest that while xLSTM-based architectures have theoretical strengths, their practical application to this specific segmentation task may be limited. Future research should investigate how to best leverage the strengths of xLSTM architectures for this specific application.

The U-Net+SA and U-Net+xLSTM models, compared to the standard U-Net, reveal smoother and more anatomically consistent ROI boundaries. In addition to the application of the segmentation results for restricting dose to the organs at risk in dose planning and general dose volume histogram (DVH) information, the generated segmentations can also serve as a basis for creating synthetic CT images. This will enable more precise dose calculations in adaptive 3D treatment planning in IOERT, especially considering the limited time available in an operative setting.

We demonstrated the feasibility of automated segmentation of CBCT data collected in an IOERT setting for breast treatment. This paves the way for image-guided adaptive treatment planning in IOERT, which is a large step towards closing the gap in dose calculation accuracy in comparison with EBRT. Even though this development has been foreseen for nearly a decade and uncertainties like tissue inhomogeneities and non-flatness of the surface have been well known [8], it has failed as of yet. This was mainly due to technical practicability, owing to the limited time in a surgical setting. The time-consuming tasks necessary for dose-volume-histogram-based planning and to compensate for inadequate image quality can be significantly shortened with the presented approach.

The current paper is limited by its relatively small dataset. This is because CBCT-guided IOERT has only recently been technically available and this is a single-centre study, which also poses a major limitation. Although more than one imaging protocol was used, no conclusion can be drawn on whether this approach can be generalized for a broad spectrum of imaging equipment and protocols. Further investigations will have to be undertaken in this regard.

## 5. Conclusions

In conclusion, this study shows that advanced neural network architectures can be successfully trained to automatically segment CBCT images acquired within an IOERT setting. By including self-attention and xLSTM features in the popular U-Net architecture, we were able to improve segmentation accuracy across critical anatomical structures, such as the lung, ribs, linac applicator, and protruding tissue. The U-Net+SA model, in particular, showed consistent improvement in Dice scores across all regions of interest, achieving an average DSC of 0.88, which surpasses the threshold of acceptability as defined in the literature [33]. The results indicate that self-attention and transfer learning help overcome the limitations of conventional U-Net models, especially in challenging imaging scenarios with artefacts and limited fields of view. The U-Net+xLSTM demonstrated limited segmentation accuracy overall, particularly for certain structures. While it did not require transfer learning, its performance variability suggests that further refinement is necessary to determine its suitability for specific applications. These findings suggest that AI-driven automatic segmentation can optimise IOERT workflows and enable adaptive 3D treatment planning. Synthetic CT images can be created from segmented CBCT data to address HU uncertainties, allowing for more accurate dose calculations and optimising the adaptive 3D planning IOERT workflow. This improvement can enable a more precise IOERT treatment within the limited time frame of surgery.

## Figures and Tables

**Table 1:** Model training parameters for the three network architectures.

Training Specifications	U-Net	U-Net+SA	U-Net+xLSTM
Learning rate	0.001	0.1	0.00005
dropout	10%	10%	40%
Activation	ReLU ^1^	ReLU ^1^	Leaky ReLU ^1^
Loss	BCE ^2^ Loss	BCE ^2^ Loss	BCE ^2^ Loss
Optimiser	Adam	Adam	Adam
Framework	Keras 2.10	Keras 2.10	PyTorch 2.0

^1^ Rectified linear unit; ^2^ binary cross-entropy.

**Table 2:** Comparison of median DSCs and standard deviations for the tube ROI over 3D CBCT across all three model architectures. U-Net+SA achieved the highest DSC values, with a significant difference (*p* = 0.002) to the standard U-Net. The U-Net+xLSTM shows lower DSC values than the standard U-Net.

Patient	U-Net	U-Net+SA	U-Net+xLSTM
Pat 1	0.88 ± 0.17	0.91 ± 0.08	0.9 ± 0.14
Pat 2	0.7 ± 0.2	0.81 ± 0.12	0.67 ± 0.2
Pat 3	0.95 ± 0.24	0.97 ± 0.09	0.88 ± 0.15
Pat 4	0.87 ± 0.14	0.96 ± 0.08	0.89 ± 0.11
Pat 5	0.76 ± 0.17	0.92 ± 0.08	0.21 ± 0.23
Pat 6	0.81 ± 0.17	0.89 ± 0.13	0.71 ± 0.26
Pat 7	0.87 ± 0.19	0.93 ± 0.07	0.82 ± 0.21
Pat 8	0.87 ± 0.22	0.9 ± 0.13	0.62 ± 0.23
Pat 9	0.78 ± 0.12	0.95 ± 0.13	0.77 ± 0.18
Pat 10	0.94 ± 0.07	0.94 ± 0.04	0.14 ± 0.27

**Table 3:** Comparison of median DSCs and standard deviations for the protruding tissue ROI over 3D CBCT across all three model architectures. U-Net and U-Net+SA achieved the highest DSC values, with no significant difference (*p* = 0.85), while U-Net+xLSTM shows lower DSC values.

Patient	U-Net	U-Net+SA	U-Net+xLSTM
Pat 1	0.78 ± 0.19	0.81 ± 0.13	0.51 ± 0.22
Pat 2	0.85 ± 0.27	0.87 ± 0.22	0.29 ± 0.12
Pat 3	0.88 ± 0.15	0.72 ± 0.16	0.47 ± 0.11
Pat 4	0.81 ± 0.13	0.77 ± 0.16	0.72 ± 0.16
Pat 5	0.66 ± 0.17	0.67 ± 0.16	0.23 ± 0.11
Pat 6	0.8 ± 0.05	0.8 ± 0.05	0.01 ± 0.08
Pat 7	0.79 ± 0.13	0.82 ± 0.11	0.13 ± 0.06
Pat 8	0.82 ± 0.06	0.8 ± 0.1	0.11 ± 0.07
Pat 9	0.7 ± 0.2	0.75 ± 0.16	0.57 ± 0.15
Pat 10	0.84 ± 0.11	0.74 ± 0.12	0.0 ± 0.04

**Table 4:** Performance metrics of the segmentation models for different ROIs. This table summarises average sensitivity, specificity, and precision with the standard deviations for the three architectures (U-Net+SA, U-Net+xLSTM, and U-Net) across four ROIs: tissue, tube, lung, and ribs.

ROI	Model	Sensitivity	Specificity	Precision
Tissue	U-Net+SA	0.83±0.09	0.99±0.00	0.72±0.11
	U-Net+xLSTM	0.35±0.23	0.99±0.00	0.54±0.25
	U-Net	0.72±0.12	0.99±0.00	0.89±0.05
Tube	U-Net+SA	0.89±0.07	0.99±0.00	0.95±0.03
	U-Net+xLSTM	0.61±0.24	0.99±0.00	0.92±0.04
	U-Net	0.78±0.10	0.99±0.01	0.93±0.06
Lung	U-Net+SA	0.94±0.02	0.99±0.00	0.97±0.01
	U-Net+xLSTM	0.88±0.09	0.99±0.00	0.97±0.03
	U-Net	0.86±0.05	0.99±0.01	0.94±0.03
Ribs	U-Net+SA	0.82±0.07	1.00±0.00	0.85±0.06
	U-Net+xLSTM	0.64±0.09	1.00±0.00	0.82±0.05
	U-Net	0.84±0.06	1.00±0.00	0.70±0.04

## Data Availability

The data presented in this study are available in this article.

## Acronyms

AI	Artificial Intelligence
CBCT	Cone Beam CT
CT	Computed Tomography
DSC	Dice Similarity Coefficient
DVH	Dose Volume Histogram
EBRT	External Beam Radiotherapy
HU	Hounsfield Units
IOERT	Intraoperative Electron Radiotherapy
SA	Self-Attention
